# Nighttime light extent and intensity explain the dynamics of human activity in coastal zones

**DOI:** 10.1038/s41598-025-85917-z

**Published:** 2025-01-11

**Authors:** Zahra Mokhtari, Angela Stefania Bergantino, Mario Intini, Mario Elia, Alessandro Buongiorno, Vincenzo Giannico, Giovanni Sanesi, Raffaele Lafortezza

**Affiliations:** 1 Department of Soil, Plant and Food Sciences, University of Bari Aldo Moro, Via Amendola 165/A, 70126 Bari, Italy; 2 Department of Economics, Management and Business Law, Laboratory of Applied Economics (LEA), University of Bari Aldo Moro, Largo Abbazia Santa Scolastica 53, Bari, Italy

**Keywords:** Coastal zone monitoring, Time series analysis, Nighttime light, Territorial sustainability, Urban ecology, Sustainability

## Abstract

Studying human activity in coastal areas is crucial for urban planning, sustainability, and economic development. However, there is limited evidence of ongoing monitoring of human activities in these areas. Thus, a quantitative analysis of the spatio-temporal changes, trends, and variability of Nighttime light (NTL) in the Italian Coastal Zone over the past decade (2014–2023) was conducted to assess human activity dynamics. The findings of our study indicate the following: (1) NTL increases over the years in both extent and intensity along the coastal zone; (2) NTL extent and intensity vary by season, with the coastal zone being brighter in summer; and (3) a highly heterogeneous NTL pattern was found where some locations became hot spots (significant upward trend) or cold spots (significant downward trend) while others remain unchanged. By explaining the intensification of human activity, this study can provide insight into identifying the patterns of economic development and environmental conditions contributing to more effective planning in coastal zones.

**Supplementary Information:**

The online version contains supplementary material available at 10.1038/s41598-025-85917-z.

## Introduction

Coastal regions represent highly populated and extensively developed urban environments, attracting migrants and tourists thanks to economic prospects, aesthetic value, and the provisioning of diverse ecosystem services and recreational facilities^1^. The expansion of urban areas and the increasing impact of anthropogenic activities are progressively reshaping coastal zones worldwide^2–4^. Human activities in coastal zones mainly include industrial, urbanization and residential development, tourism and recreation, transportation, fisheries, and agriculture, resulting in changes to territory, society, and the economy^5^. Understanding the dynamics of urbanization and human activity is crucial for promoting more sustainable human settlements in coastal zones^5,6^. However, little statistical evidence refers to this specific aspect, since official statistics, which often refer to administrative borders and the direct collection of data through surveys, are quite expensive. The lack of precise measurements of human activity along the global coastal strip presents a clear data gap that hinders effective planning and development strategies for coastal and inland regions^6^.

Although mapping land use/land cover (LULC) changes by daytime satellite images (e.g., from Landsat, Sentinel, and MODIS products) is a commonly used tool to investigate urban dynamics and to identify physical changes in built-up areas, these images are not able to capture the degree of urban intensity and human activities in cities^7^. In addition, LCLU data are often collected at specific intervals (i.e., annually) and thus pose a limitation to monitor rapid changes (e.g., disaster recovery and seasonal tourism peak)^8^. In recent decades, remote sensing of Nighttime light (NTL) has become a reliable proxy to investigate human-related activities connected to detect alterations in demographics, the economy, energy consumption and urban extent^9–20^. “NTL extent” and “NTL intensity” are two measurements that can track the human footprint in urban areas. NTL extent refers to the spatial coverage of the bright area resulting from changing physical built-up area, while NTL intensity is associated with concentration or magnitude of light sources^21,22^. The NTL dataset derived from Visible Infrared Imaging Radiometer Suite (VIIRS), located on-board the Suomi National Polar-orbiting Partnership (SNPP) satellite, is widely used to investigate urbanization and human activities due to its high temporal (i.e., monthly data) and spatial resolution and sensitivity to visible light^23^. NTL imagery is useful for analyzing the nighttime economy (e.g., active tourism areas, industrial operations, and infrastructure development) which is not visible during the day, offering unique insights that multispectral LCLU data cannot provide^8^.Therefore, it is considered suitable for quantifying the spatio-temporal dynamics of human activity intensification in a cost-effective manner^20^, especially in regions that are influenced by light-based seasonal human activities^8^ like coastal regions. Therefore, combining both daytime and nighttime provides a comprehensive view of urban dynamics and human activities^8^.

The role of NTL datasets becomes even more critical with the lack of reliable official statistics^17^ to detect and estimate socioeconomic dynamics at a specific spatial (i.e., subnational and non-administrative unit) and multi-temporal level^21^. Temporal fluctuations of NTL intensity allow researchers to track the intensity of population displacement resulting from disaster occurrences or cultural celebrations, holidays, and tourist populations^8^. The availability of multi-temporal NTL datasets provides the opportunity to extract time series data and analyze trend changes, enabling the investigation of various aspects of human activities in settlement development^24,25^ and the assessment of economic activity or tracking of population movements^17,26^ in a timely and spatially explicit manner. For instance, Stathakis and Baltasthe^26^ utilized multi-temporal VIIRS nighttime datasets as a proxy to estimate the seasonal population in Greece. They discovered a strong correlation between NTL and tourist population intensity. Yun et al.^27^ assessed socioeconomic development across Tiwan Strait from 2000 to 2020. They found out that the urbanization level in coastal areas is higher than that in inland areas. Pambuku et al.^28^ employed temporal variation of NTL imagery to investigate the impact of the tourist populations in the coastal region of Puglia, Italy. Their findings showed that increasing NTL intensity is correlated with tourism population influx in this region. Despite the critical importance of human activity dynamics in coastal zones^29^, studies focusing on how these activities fluctuate over time and across different seasons in coastal areas are still limited. Several statistical models are available that allow researchers to investigate and visualize the trend of changes, shifts and variability of environmental phenomena. However, these tools have rarely been used to investigate trends of change in NTL remote sensing particularly in coastal areas.

The Italian Coastal Zone, the focus of this investigation, is one of the most popular tourist destinations being affected by various and intense human activities such as urbanization, industrial construction, and touristic exploitation^30^, but where dependable statistics related to human activity are scant. The overarching goal of this research is to assess the spatio-temporal change of NTL extent and intensity in the Italian Coastal Zone as a proxy to estimate human activity from 2014 to 2023. Due to the uneven environmental and economic statuses in Italy^31,32^, the NTL rate of change is hypothesized to be different in the regions of the North, Center, and South. Since many human activities like tourism are seasonal in nature^8^, we also assume that seasonality influences the extent and intensity of NTL. Therefore, the objectives of this study are: (1) To investigate the dynamics of NTL over the past decade (2014–2023); (2) To explore the trend and magnitude of variability in NTL intensity across different regions—North, Center, and South; (3) To examine the impact of seasonality on the extent and intensity of NTL in various parts of the coastal zone; and (4) To visually depict the spatial and temporal shifts and variability of NTL over the past decade.

## Materials and methods

### Study area

The focus of this study is on the entire Italian Coastal Zone (including the islands of Sicily and Sardegna), which borders the Adriatic, Ionian, Tyrrhenian, and Ligurian Seas^33^. Italian coastal zones are densely occupied by residential settlements and characterized by considerable economic activities such as tourism as well as road and sea transport infrastructures^34^. According to ISTAT^33^, 16.8 million inhabitants permanently live in the 642 coastal municipalities, encompassing about 30% of the total population.

**Fig. 1:**
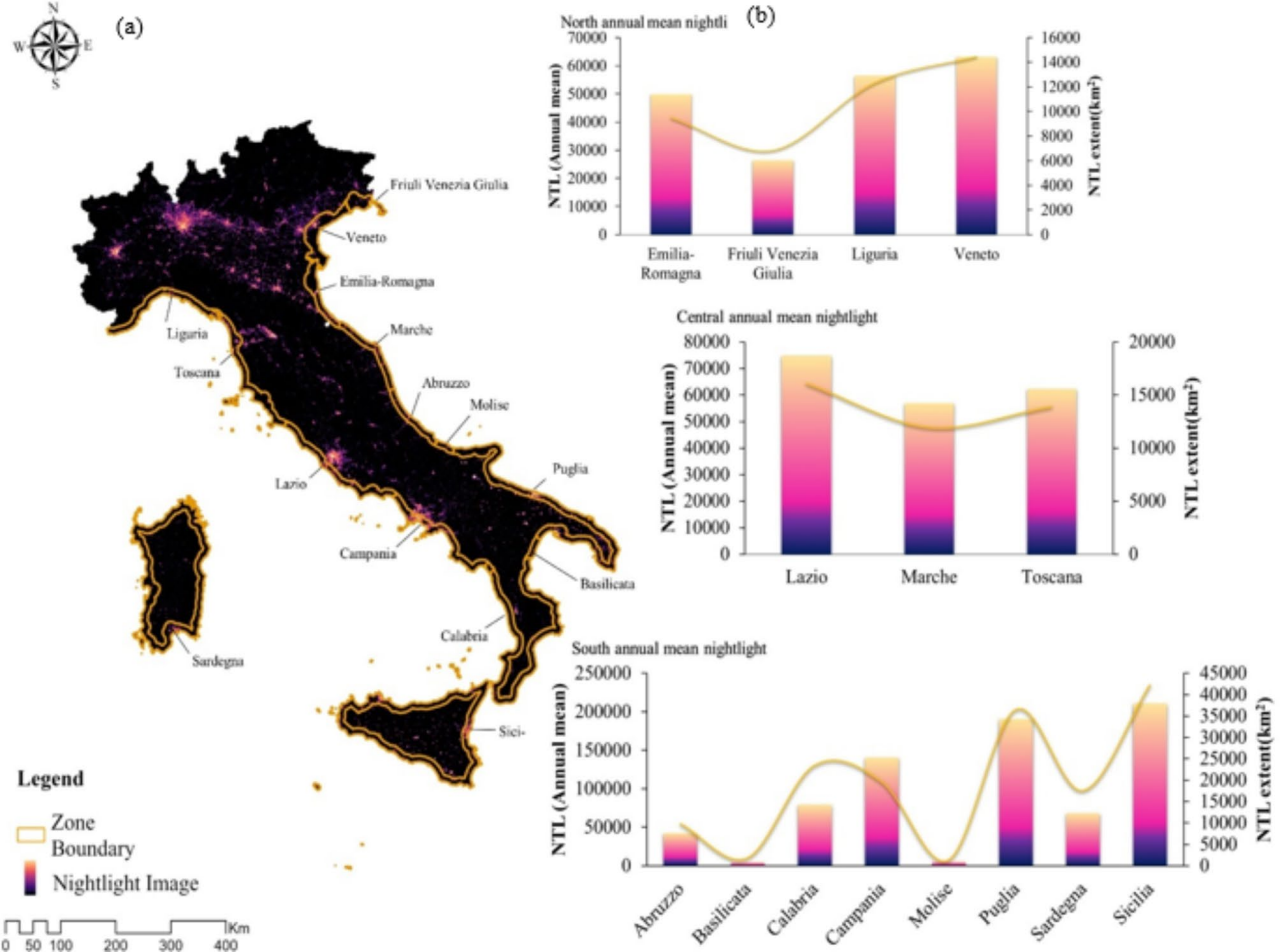
(**a**) Nighttime light (NTL) image in the Italian Coastal Zone from VIIRS Stray Light Corrected Nighttime Day/Night Band Composites Version 1, Google Earth Engine^38^ created in ArcGIS Pro 3.3 (https://pro.arcgis.com/en/pro-app/latest/get-started/release-notes.htm); (**b**) Annual average NTL intensity for each province in the North, Center, and South of Italy in 2023.

In order to frame the boundary of the study area, the Italian coastline was defined by an inland depth of 10 km^34^ (Fig. 1). The 10-km buffer zone is often used in coastal zone studies for consistency and to capture a wide range of effects, facilitating the comparison of results across different regions or studies^37,38^. Furthermore, a 10-km buffer can encompass the main tourism infrastructure (hotels, resorts, transportation), providing a better understanding of the human activity and economic impact on the coastal zone.

Italy is a country marked by dramatic structural and economic contrasts across different regions. Italian regions are remarkably different in terms of demographic pattern, economic development, social affluence, and institutional quality^32^. Moreover, Italy is commonly divided into three major geographical regions: North, Center, and South^33^. This classification is essential in economic studies since it reflects the economic, industrial, and social distinctions within the country. Using this division, economists and policymakers are enabled to frame discussions to compare regional development and inequality^37^. In this study, the Italian Coastal Zone was analyzed distinctively using the classification scheme North, Center, and South (Fig. 1).

### Built-up land cover provision

We investigated the change of physical expansion of built-up area as a reference to define and validate the NTL extent. Then, we extracted built-up area from 2015 to 2023 using Dynamic World, which provides high-resolution (10 m) near-real-time land cover data derived from satellite imagery collected by the European Space Agency’s Sentinel-2 satellites^39,40^ (the built-up area for 2014 was not available in this dataset). In terms of accuracy, Dynamic World continuously assesses land cover predictions by comparing them with reference datasets and using common accuracy metrics such as overall accuracy, user’s accuracy, and producer’s accuracy. The overall accuracy of the Dynamic World classification is 70.8–82.1%^41^.

### NTL collection and processing

The monthly VIIRS NTL composite data, derived by the Earth Observation Group in the Colorado School of Mines^42^, from January 2014 to September 2023, were downloaded from the Google Earth Engine platform. VIIRS NTL data are available in GeoTIFF format, which can be opened with GIS software e.g., QGIS. The spatial resolution of the dataset is 15 arc-seconds (~ 500 m) with a unit of nano-W cm^− 2^ sr^− 1^. This product is an alternative configuration of the VIIRS Day Night Band (DNB) in which a procedure is used to correct stray light^43^. The main characteristics of the NTL data are shown in Table 1.

Because many human activities are seasonal in nature^8^, the temporal resolution of the analysis is monthly, so that seasonal patterns can be identified^26^. Summer-based NTL is the average of the hot season including June, July, August, and September, while the winter NTL considers the average values for the cold months of December, January, February, and March.

**Table 1:** Main characteristics of the VIIRS nighttime light data.

VIIRS nighttime light image	Characteristics
Spectral band	1 panchromatic
Radiometric resolution	12 bits, values in (0.4096)
Temporal resolution	1 month
Spatial resolution	463.83 m
Availability	2012-present (uncorrected) 2014-present (stray corrected)
Unit	nW cm^− 2^ sr^− 1^

*VIIRS* Visible infrared imaging radiometer suite.

According to the pre-processing method proposed by Li et al.^44^, we filtered the dark background and eliminated the abnormal NTL intensity. Once a pixel’s NTL intensity is lower than 1 nano-W cm^− 2^ sr^− 1^, this pixel is considered as dark background mask and removed from the extent. In addition, following the method by Li et al.^44^, the pixels of NTL which were higher than the maximum NTL value in the center of the main urbanized area were recognized as abnormal and removed from the dataset.

### NTL extent

Identifying the NTL extent by testing thresholds can decrease or eliminate its blooming effects (overestimation of bright urban areas)^8,45^. The blooming effects of the data can make the dim regions to be seen as bright areas on satellite images^45^. Following the main research in this area by Shi et al.^46^, we tested values between 5 and 10 nanowatts/cm²/sr using the reference built-up land cover class by Dynamic World (2023) to ensure that the chosen threshold delineates built-up areas. In our study, the threshold value was found to be approximately 5 nanowatts/cm²/sr to capture built-up area.

To calculate the extent growth rate from 2014 to 2023, the following formula was used:

1 $$\:\left(\right(\:{NTL\:Extent}_{2023}-{NTL\:Extent}_{2014})\:/({NTL\:Extent}_{2014}\left)\right)\times\:100$$

To investigate if the seasonality affects the extent, the NTL extent growth was calculated for the winter and summer seasons.

### NTL intensity

In this study, we used Sum of Lights (SoL) which represents the total brightness or radiance values recorded over a given spatial unit (Eq. 2)^22^. SoL can effectively reflect the concept of human activity intensity instead of the mean NTL data that were used to simulate human activity intensity in this study^8^.

2 $$\:SoL\:=\sum\:_{i=1}^{n}{Radiance}_{i}$$

Radiance is the value of the *i*th pixel, and *n* is the total number of pixels in the given area.

### Trend analysis of NTL intensity

Trend analysis was employed to identify significant trends in a time series (2014–2023) of NTL datasets. This study utilized the non-parametric methods of MK Significance Trend Test and Sen’s Slope Estimator to assess NTL trend over the past decade. Sen’s Slope method, a non-parametric statistical approach, was employed for time series analysis to determine the increasing or decreasing magnitude of the trend in a series of datasets^47^. Sen’s Slope unit represents the magnitude of the slope per year or per month. The MK Significance Trend Test is a quantitative analytical tool utilized to evaluate the significance of changing trends in time series datasets. In comparison to parametric testing methods that necessitate data normality, the MK method is considered a suitable test for discerning data trends and ranking the observed values along with their position in the time series data^48^. Typically, the combination of Sen’s Slope method and MK Significance Trend Test is capable of evaluating the trend and its magnitude in a time series dataset^47^.

In this research, the sequential input is seasonal and monthly NTL intensity from 2014 to 2023. The Null Hypothesis defines that there is no trend in the time series data, while the Alternative Hypothesis suggests that an increasing or decreasing trend can be seen over time. A typical significance level (α) used is α = 0.05 (5%) that defines the threshold for determining if the observed trend is statistically significant. The details of the parameters for this statistical test are provided below.

#### Mann–Kendall significance trend test

The significance of the trend was examined using the Mann–Kendall (MK) Significance Trend Test^49^. The MK test is calculated as follows:

3 $$\:S=\sum\:_{i=1}^{n=1}\sum\:_{j=i+1}^{n}sgn\left(NTLi-NTLj\right)$$

where *n* is the number of data points, *xi* and *xj* are the NTL values in the time series *i* and *j*, respectively, and *sgn (xj − xi)* is the significance function as:

4 $$\:sgn\:\left({NTL}_{i}-{NTL}_{j}\right)=\left\{\begin{array}{c}+1\:\:if\:NTLj-NTLi>0\\\:0\:\:\:\:if\:NTLi-NTLj=0\\\:-1\:if\:NTLi-NTLj<0\end{array}\right.$$

where *NTLi* and *NTLj* are the *NTL* amount at time *i* and time *j*, respectively. The variance *δ (S)* is defined as:

5 $$\:\delta\:\:\left(S\right)=\frac{n\:(n-1)(2n+5)}{10}$$

6 $$\:\delta\:\:\left(S\right)=\frac{n\:(n-1)(2n+5)}{10}$$

The Z-values are calculated as follows:

7 $$\:z\left\{\begin{array}{c}\frac{S-1}{\sqrt{\delta\:\:\left(S\right)}\:}\:S>0\\\:0\:S=0\\\:\frac{S+1}{\sqrt{\delta\:\:\left(S\right)}}\:S<0\end{array}\right.$$

The trends were tested at a specific α significance level. Positive *Z* values indicate increasing trends while negative *Z* values reflect a decreasing trend. The MK Significance Trend Test results at a significance level of α = 0.05 (95% confidence level) and can be categorized as a significant downward trend (*S* < 0 and |Z| > 1.96), weak downward trend (*S* < 0 and |Z| ≤ 1.96), no trend (*S* = 0), weak upward trend (*S* > 0 and |Z| ≤ 1.96), and significant upward trend (*S* > 0 and |Z| > 1.96)^44^.

#### Sen’s slope estimator

Sen’s Slope Estimator^50^ was used to calculate trends in NTL time series:

8 $$\:\text{S}\text{N}\text{T}\text{L}\:=\:Median\:\left(\frac{{NTL}_{j}-{NTL}_{i}}{j-i}\right)$$

where S_NTL_ is Sen’s Slope Estimator, showing the trend in the NTL time series; *Median* is the function that considers the median of the slopes of all lines; *i* and *j* are time series numbers; and *NTLi* and *NTLj* are the NTL value at time *i* and time *j*, respectively. When *S*_*NTL*_*>* 0, the NTL time series shows an increasing trend, *S*_*NTL*_ ˂ 0 indicates a decreasing trend, and *S*_*NTL*_ = 0 shows an unchanged trend.

### Spatial pattern of pixel-based Z-value

To illustrate the level of change, we divided the Z-scores resulting from the MK test into five classes showing significant and weak downward, upward and no significant change for each pixel (see the classification in the section of Mann–Kendall Significance Trend Test). To illustrate the Z-value pattern across the study area, the Python package “pyMannKendall1.4.3”^51^ was used, as it offers all types of MK tests and enables the researcher to analyze and illustrate MK trends^52^.

### Emerging hot spot analysis

The Emerging Hot Spot Analysis illustrates changing trends in time series data^53^. It calculates clusters of NTL to identify the aggregation, or clustering degree, of the high value (hot spot) and low value (cold spot) of the spatial variable^53,54^. The analysis results indicate the locations with statistically significant upward or downward trends for hot/cold spot Z-scores across 17 classes. Emerging Hot Spot Analysis was performed using ArcGIS Pro’s Space Time Pattern Mining toolbox. The tool allows to detect spatial clusters of NTL intensity changes over time. The seasonal (winter and summer) NTL data from 2014 to 2023 were used as the primary input for the analysis. The neighborhood distance or spatial relationship for hot spot detection was defined based on the “Spatial Autocorrelation by Distance”. The analysis used a Z-score 95% confidence level (p < 0.05) to identify statistically significant clusters of changes in NTL intensity.

### Accuracy assessment

For the accuracy assessment of NTL data (illuminated and non-illuminated area) a land cover data (built-up area and non-built-up area). In order to match spatial resolutions, we resampled NTL. A pixel-based overlay analysis was performed to compare NTL points with land cover, and a confusion matrix was generated to compute accuracy metrics.

## Results

### NTL extent growth rate

The growth rate of NTL extent was calculated in both winter and summer. As shown in Table 2, the North region experienced a modest growth rate of 3 and 6%, respectively, in winter and summer, while the South showed consistent growth in both seasons, especially in summer, with a 10.7% increase. Overall, the total NTL extent increased by 4.3% in winter and 9.2% in summer, indicating more pronounced urban growth or increased nighttime activity in summer months across the regions.

**Table 2:** Nighttime light (NTL) extent growth rate in the Italian Coastal Zone (2014–2023).

	NTL extent (km^2^) in winter			NTL extent (km^2^) in summer		
	2014	2023	% Growth	2014	2023	% Growth
North	1941.5	1999.8	3	1952	2070	6
Center	1925.8	1936.0	0.5	1958.6	2107.2	7.5
South	6348.0	6720.4	5.8	6854.8	7588.8	10.7
Total	10216.4	10656.5	4.3	10766.2	11766.7	9.2

### Trend analysis of NTL

#### Sen’s Slope Estimator and MK significance Trend Test on seasonal NTL

**Fig. 2:**
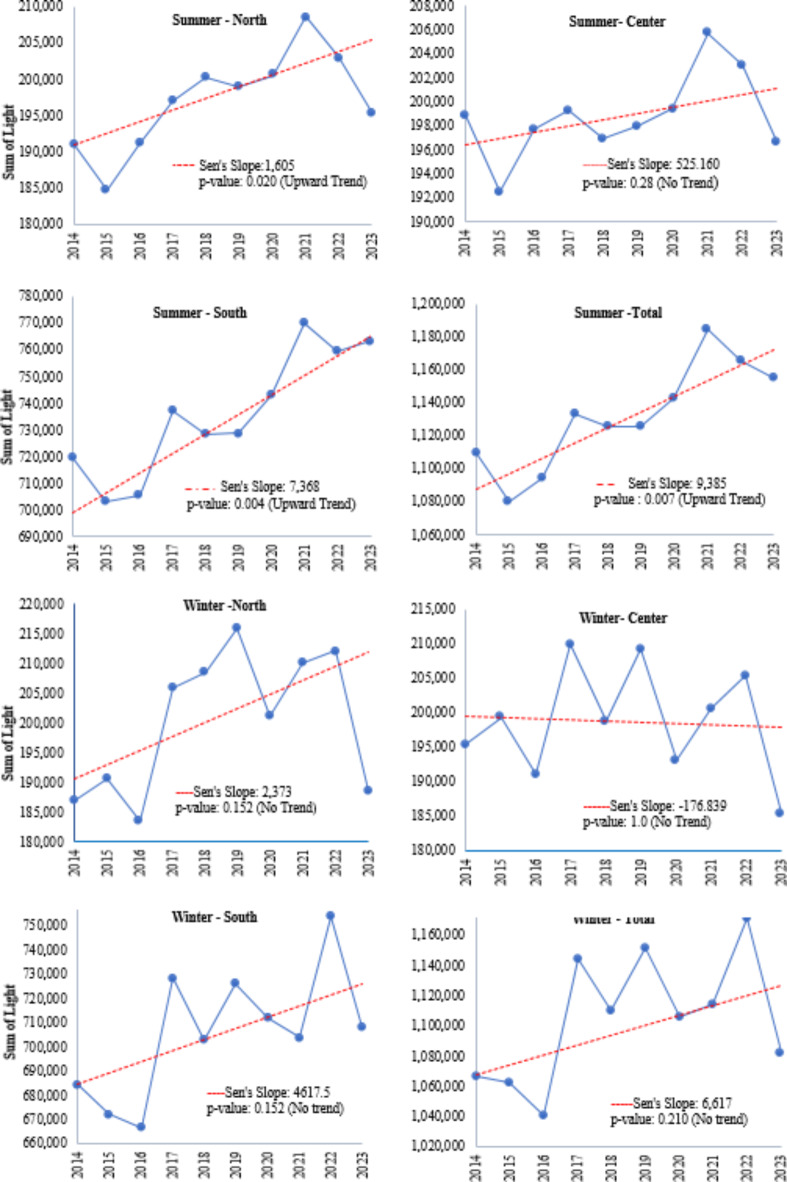
Temporal change of Nighttime light (NTL) using Sen’s Slope Estimator and Mann-Kendall Significance Trend Test on seasonal NTL (winter and summer) in North, Center, and South and total coastal zones (2014–2023). *Dotted red line indicates the magnitude of trend based on Sen’s Slope Estimator.

SoL was calculated in the two seasons winter and summer in North, Center, and South for 10 years from 2014 to 2023. As shown in Fig. 2, the NTL value in the South was higher than in the other regions because it covered a larger area.

The results of the MK Significance Trend Test indicated a significant upward trend in summertime, particularly in the South (Sen’s Slope = 7368), with the Center showing some exceptions. Conversely, no significant trend was observed in the winter season across the coastal zone. Although there was an overall increase in the summertime trend over the last decade, the magnitude of the change varied from year to year. The most noticeable change occurred after a sharp increase in NTL from 2020 to 2021 during the Covid pandemic. Following this event, there was a sharp decrease in NTL in 2022 for all parts of the coastal zone except the South, which showed a less dramatic decrease. In 2023, the South recorded a higher NTL value compared to other parts of the coastal zone. Also, for NTL time series values, the wintertime trend was related to the lower NTL in 2020 compared with the same value in 2019 and 2021 (Fig. 2).

**Table 3:** Mann–Kendall trend test.

	Kendall’s Tau		Sen’s Slope		*p*-value (two-tailed)		Alpha
	Winter	Summer	Winter	Summer	Winter	Summer	
North	0.378	0.6	2373.9	1605.5	0.15	0.020*	0.05
Center	0.022	0.289	− 176.8	525.16	1.0	0.283	0.05
South	0.378	0.733	4617.5	7368.5	0.16	0.004*	0.05
Total	0.33	0.689	6617.8	9385.9	0.21	0.007*	0.05

*Significant trend observed.

Table 3 shows statistical results comparing the winter and summer trends across three regions (North, Center, and South) and the total for Kendall’s Tau correlation, Sen’s Slope, and the two-tailed p-value, with an alpha significance level of 0.05. According to p-value, the trend is significant in Summer (North, South and Total).

### The spatial pattern of Z-value

**Fig. 3:**
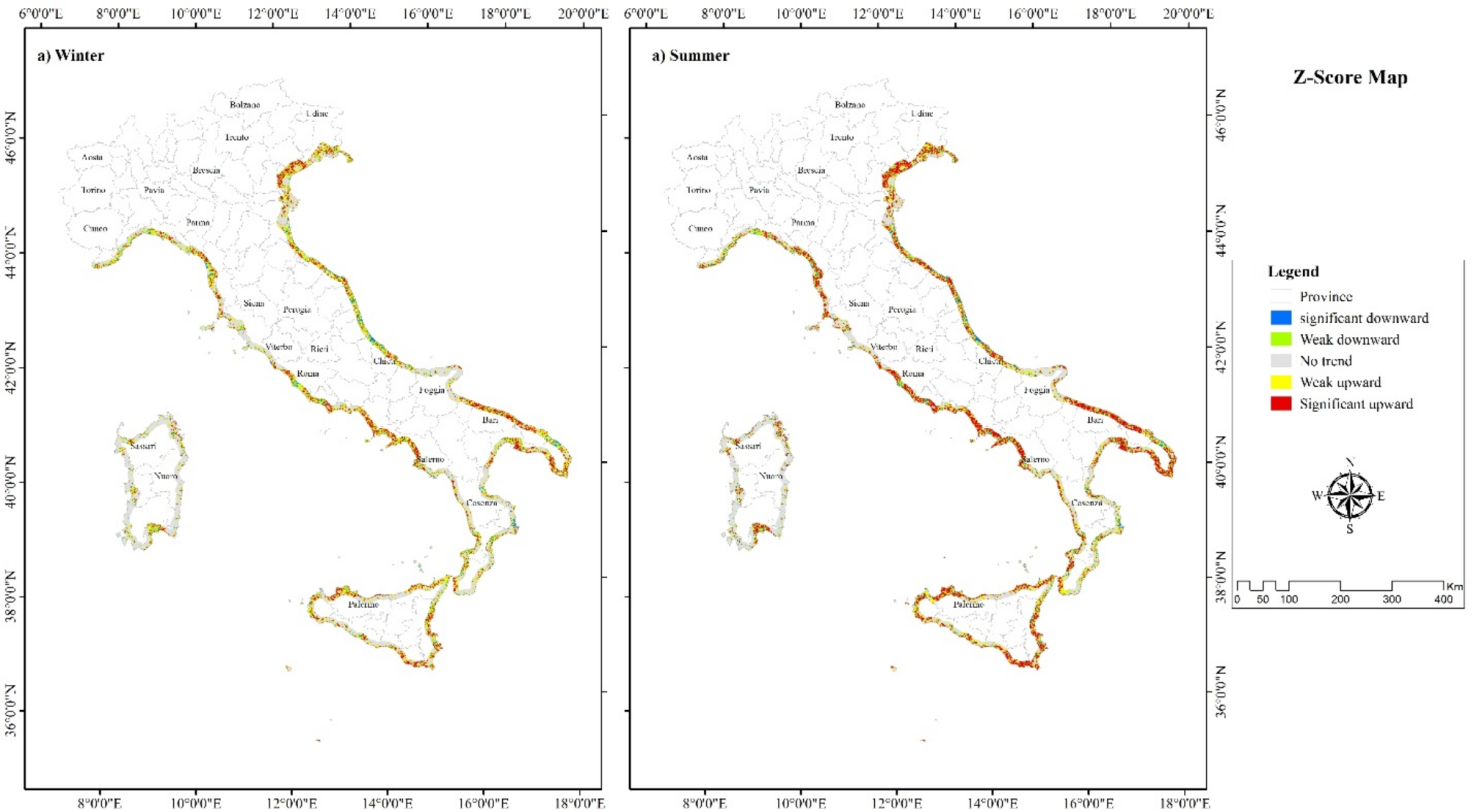
The spatial pattern of trend (2014–2023) using package “pyMannKendall1.4.3” available at: https://github.com/mmhs013/pyMannKendall in (**a**) winter and (**b**) summer.

The spatial pattern of the pixel-based Z-values resulting from the MK trend illustrates whether the location showed an increasing, decreasing, or unchanged trend. As depicted in Fig. 3, the Z-value pattern was heterogeneous throughout the study area, particularly in the South. The significant downward, weak downward, unchanged, weak upward, and significant upward trend classes accounted for 5%, 10%, 36%, 20%, and 26%, respectively.

### Emerging hot spot analysis

**Fig. 4:**
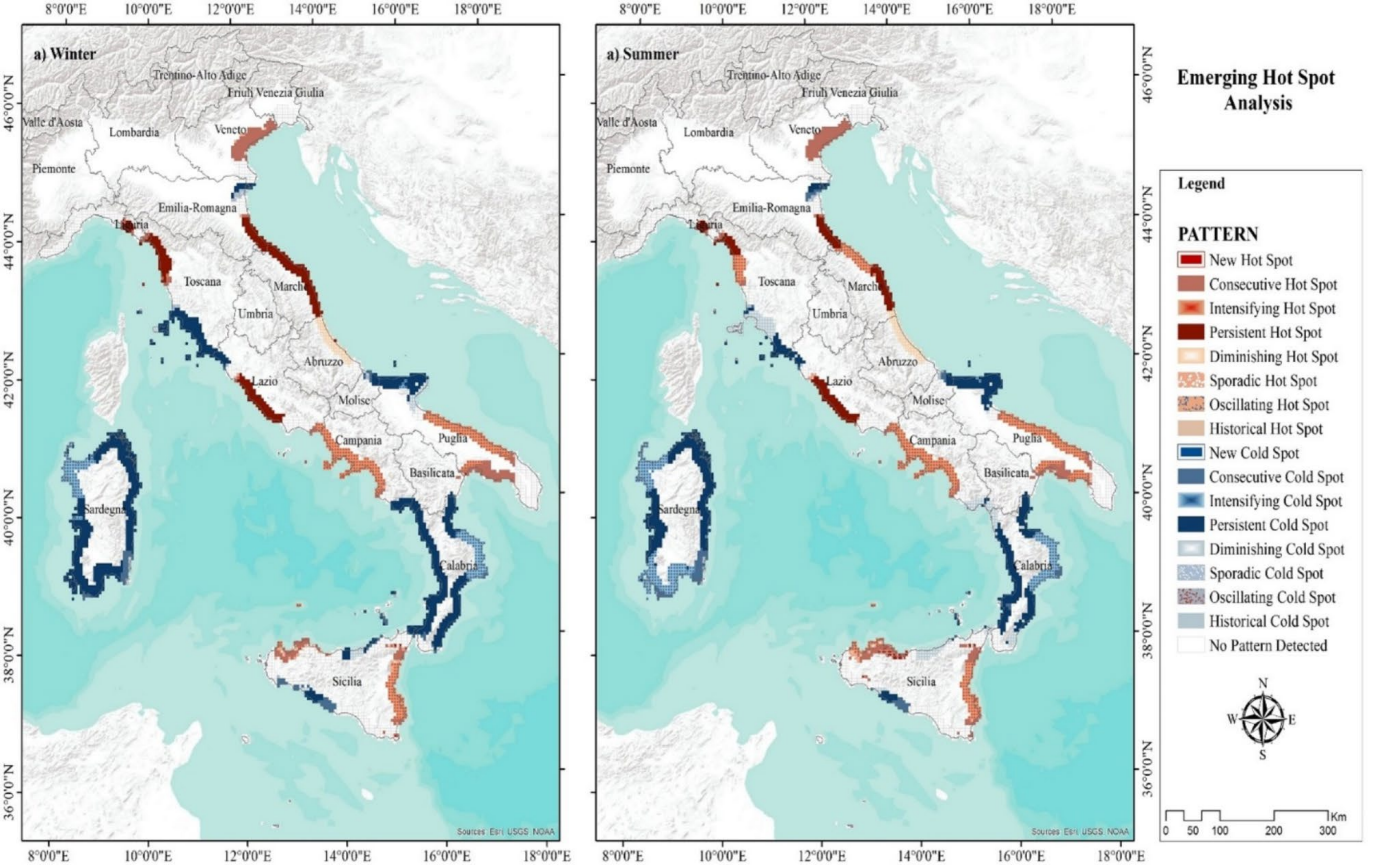
Classification of NTL variation trend (2014–2023) using the Emerging Hot Spot Analysis, ArcGIS Pro 3.3 (https://pro.arcgis.com/en/pro-app/latest/get-started/release-notes.htm).

The Emerging Hot Spot Analysis or “Space Time Pattern Mining” results categorize the trend of NTL intensity into a variety of classes (Fig. 4). The analysis revealed a heterogeneous pattern throughout the coastal area, with some locations identified as hot spots or cold spots, while certain areas remained unchanged over the last decade. Although the analysis classified the area into 17 classes, for the purpose of this study we only focused on locations with intensifying hot/cold spots. The analysis highlighted several significant findings in various regions: Puglia and Campania have emerged as intensifying hot spots over the last 10 years, indicating a significant increase in the high cluster of NTL. Sardegna and Calabria exhibited a mixture of persistent and intensifying cold-spot patterns, characterized by low NTL clusters over time. Sicily demonstrated a heterogeneous pattern, with the southeastern part identified as an intensifying and persistent hot spot over the last decade. The coast of the Basilicata region remained largely unchanged. Abruzzo and Marche formed an almost continuous hot spot in the Center. In the North, the pattern remained almost unchanged (Fig. 4).

### Accuracy assessment

An accuracy assessment was conducted using 72,482 validation points (30% of total points) of the NTL points were used randomly within the defined study area. Among these, 6355 points were classified as built-up areas in both the NTL and land cover datasets, while 13,398 points identified as illuminated in the NTL dataset were classified as non-built-up in the land cover dataset. Additionally, 52,148 points were correctly identified as non-built-up in both datasets, whereas 481 points were misclassified as built-up in the land cover dataset but were non-illuminated in the NTL dataset. The overall accuracy of the classification was 80.9%, indicating good agreement between the datasets (Table 4).

**Table 4:** Metrics of accuracy assessment.

Metric	Value
Producer’s accuracy (built-up)	32.2
Producer’s accuracy (non-built-up)	99%
User’s accuracy (built-up)	92%
User’s accuracy (non-built-up)	79%
Total accuracy	80%
Kappa	0.54

## Discussion

### Time series analysis and spatial pattern of NTL

The study analyzed the spatio-temporal changes in Nighttime light (NTL) extent and intensity to evaluate human activity dynamics in the Italian Coastal Zone from 2014 to 2023. In order to define the threshold of built-up area and assess the accuracy, we extracted built-up class from World Dynamic^39^ datasets. The findings indicated an overall increase in both the extent and intensity of NTL during this time period, with varying rates of increase and variability across different coastal zones. Notably, the South exhibited a higher NTL extent growth rate and a more pronounced upward trend. The pixel-based Z-score pattern of the Mann–Kendall Significance Trend Test revealed that 26% of the total coastal area showed a significant increasing trend, while 20% exhibited a weak increasing trend. This trend in human-induced urban sprawl is believed to be largely driven by mass tourism increase in coastal destinations^29,55^. Generally, the tourism industry leads to infrastructure development (i.e. roads, railways, utilities network) and land use/land cover change^56^.

The seasonal peaks of human activity depend on the location that attracts people for a particular purpose^8^. Intense human activities in coastal areas during the summer season are associated with tourist populations^26^. Similarly, several studies found that a seasonal tourist activity leads to an increase in the number of tourist accommodations and services in Mediterranean coastal cities^28,57^. In our research, we analyzed the growth rate and intensity of NTL during two seasons, summer, and winter, to investigate potential differences in human activities. Our findings revealed that the growth rate of NTL (2014–2023) was higher in summer than in winter across the coastal zone. However, in the South, more dim areas are transformed into bright areas in summer. The results of the Mann–Kendall Significance Trend Test indicated that the trend did not significantly increase in winter, while an upward trend was observed during the last decade in summer. Our research also indicated that the coastal area experiences seasonality, with the Italian Coastal Zone being brighter in summer than in winter. Additionally, the Covid-related lockdown has affected the NTL values. This impact is evident when comparing the NTL value in winter 2020 with the values in 2019 and 2021 (Supplementary Fig. S2). Moreover, the decrease in NTL values across the entire coastal zone in 2023 may be attributed to economic pressures, such as oil prices and transportation costs, which have been influenced by the conflict between Russia and Ukraine that began in February 2022.

In this research we used the Dynamic World dataset^40^ for detailed monitoring of changes in built-up areas over time, providing a robust foundation for our analysis of urban expansion and identification of NTL extent. The results showed an increasing extent of bright areas in coastal zones (Table 2), which aligned with the increase in built-up area extracted from the Dynamic World datasets (Supplementary Fig. S1). In addition, according to the Coastal Port report, the Italian coastal area is changing due to anthropogenic activities, such as defense, port facilities and tourist use, in such a way that natural and agricultural land is being replaced by built-up areas^58^.

To illustrate changes in NTL intensity over time, we applied the variation trend of pixel-based Z-value pattern and Emerging Hot Spot Analysis. The resulting map categorized NTL hot spots into 17 classes, showing statistically significant and weak upward, downward, or unchanged trends. The trend of change and variability in the coastal zone revealed a heterogeneous pattern, especially in the South. In the South, the Puglia and Campania regions were classified as intensified hot spots, whereas Calabria and Sardegna were classified as intensifying cold spots over the last decade. Similarly, Pambuku et al.^28^ showed that there was an increasing trend of NTL and the tourist population over the past decade in the Puglia region, Southern Italy^28^. Moreover, the higher growth rate of light expansion in the South is consistent with the findings of Hjalager^59^, according to which the highest percentage of illegal construction in the Italian coastal zone occurred in the southern parts. This means that the south has been more affected by human activities in the last 10 years.

### Policy implications

The rise in NTL in coastal zones in Italy is attributed to increased urbanization, economic activity, and population expansion. While this may indicate economic growth, it also raises concerns about environmental degradation, resource exploitation, and vulnerability to climate change and natural hazards^60^. Policy regulation needs to include stricter norms on zoning laws to restrict construction in high-risk areas, manage urban sprawl and reduce environmental impacts. In addition, NTL growth may occur in tandem with the Urban Heat Island (UHI) effect, which contributes to higher temperatures, increased energy demands, and public health risks^61–63^.

To mitigate these impacts, policymakers must prioritize sustainable urban planning that includes green infrastructure, energy-efficient building, and climate-resilient designs. For example, in this study, the coastal region of Puglia, which has experienced intensified human activity, may face environmental challenges like improper waste disposal and increasing energy consumption. By integrating NTL data into urban planning, decision makers can address the challenges posed by the UHI effect and enhance cities’ long-term resilience to climate change. Conversely, the regions of Sardegna and Calabria with decreasing NTL trends may indicate economic decline or depopulation. While this could imply a reduction in human impact, it may also signal an economic recession. In these cases, with decreasing NTL, policies should focus on improving coastal economies by promoting sustainable tourism or investing in small businesses.

### Limitations and future perspectives

In this research, we considered only light-based human activities which do not represent the activities occurring in low-light areas without electricity, for example, agricultural-related activities. In general, like all remotely based methods, using NTL as a proxy for estimating human activity is associated with several uncertainties which arise from data characteristics and analytical methods. It should be noted that most of the light detected by NTL sensors is from upward light emissions, whereas the inside lighting of buildings cannot be variably detected^8^. This inconsistency may lead to an underestimation of the intensity of human activity in the study area. Further, some streetlights may turn off early at night, a factor which affects nighttime brightness data^64^. In addition, the spectra or radiance of artificial light emissions may be radiated differently due to different lamp colors, types, or spectra of illumination^8^. In addition, due to poor resolution, VIIRS/DNB cannot capture LED light sources with short-wavelength peaks^65^.

As far as the limitations of the methodology are concerned, the Mann–Kendall test is generally robust in analyzing the trends of various datasets, however, extreme outliers can affect the results, especially in small samples^66^. In addition, various methods are available to find NTL thresholds that detect bright extent, but the results may be accompanied by some degree of uncertainty^23,67^, especially in trend analysis.

Future studies should focus on acquiring NTL data from multiple sources to obtain a long-term NTL series using different fusion techniques. Such studies can also investigate the change in NTL data in the coastal zone compared with the interior area to better illustrate how the coastal area is being altered by human activities. Lastly, the relationship between NTL data and social and environmental indicators can be explored to investigate their effects on NTL intensity and human activities.

## Conclusions

In the present study, time-series analysis of NTL remote sensing imagery was used to compensate for the lack of statistical data on human activities in coastal areas. We employed Sen’s Slope Estimator, the Mann–Kendall Significance Trend Test, and Emerging Hot Spot Analysis to analyze the trends and distribution patterns of NTL as a proxy for urbanization and human activities in the Italian Coastal Zone over the last decade. Our results indicate that from 2014 to 2023, the extent and intensity of NTL increased along the coastal zone, with approximately 46% of the coastal area experiencing an increasing trend in NTL over the last 10 years. The increase was more pronounced in the South than in the North. As expected, the coastal area was brighter in summer compared to winter. The Emerging Hot Spot Analysis revealed a spatially heterogeneous pattern of NTL dynamics over the last decade, most remarkably in the South, with Puglia and Campania classified as intensifying hot spots and Sardegna and Calabria as intensifying and persistent cold spots of NTL.

These findings can provide an evidence-based holistic portrait of the Italian Coastal Zone in relation to human activities that can assist policy makers to make data-driven decisions. The growth of NTL intensity and extent is a double-edged sword—it is economically promising; however, it is likely to have a destructive impact on the regional environment, especially with regard to the effects of climate change in the coastal regions of the Mediterranean basin. Therefore, there is an urgent need for integrative planning that considers economic and environmental aspects to promote inclusive, sustainable, and resilient development in the Italian Coastal Zone.

## Electronic supplementary material

Below is the link to the electronic supplementary material.

Supplementary Material 1

Supplementary Material 2

## Data Availability

The data used in this paper are available from the corresponding author upon reasonable request.
