# Supplementary material for: Nighttime light extent and intensity explain the dynamics of human activity in coastal zones
Source: Sci Rep. 2025 Jan 11;15:1663. doi: 10.1038/s41598-025-85917-z (PMC11724959; doi:10.1038/s41598-025-85917-z)
Supplement: Supplementary file 1 — Supplementary Material 1 [file 41598_2025_85917_MOESM1_ESM.pdf]

Nighttime light extent and intensity explain the dynamics of human activity in coastal zones

Zahra Mokhtari[a], Angela Stefania Bergantino[b], Mario Intini[b], Mario Elia[a], Alessandro Buongiorno[b], Vincenzo Giannico[a], Giovanni Sanesi[a], Raffaele Lafortezza[a,c*]

Supplementary material

1. Built-up land cover change

We obtained the Built-up cover for 2015 and 2023 from Dynamic World datasets. The extent of the built-up class expanded by 5% over this time frame. The comparison of built-up areas in 2015 and 2023 indicated that the rate of increase of new built-up patches in the South was about 5%, which is higher than in the northern (1.3%) and central (2.3%) regions.

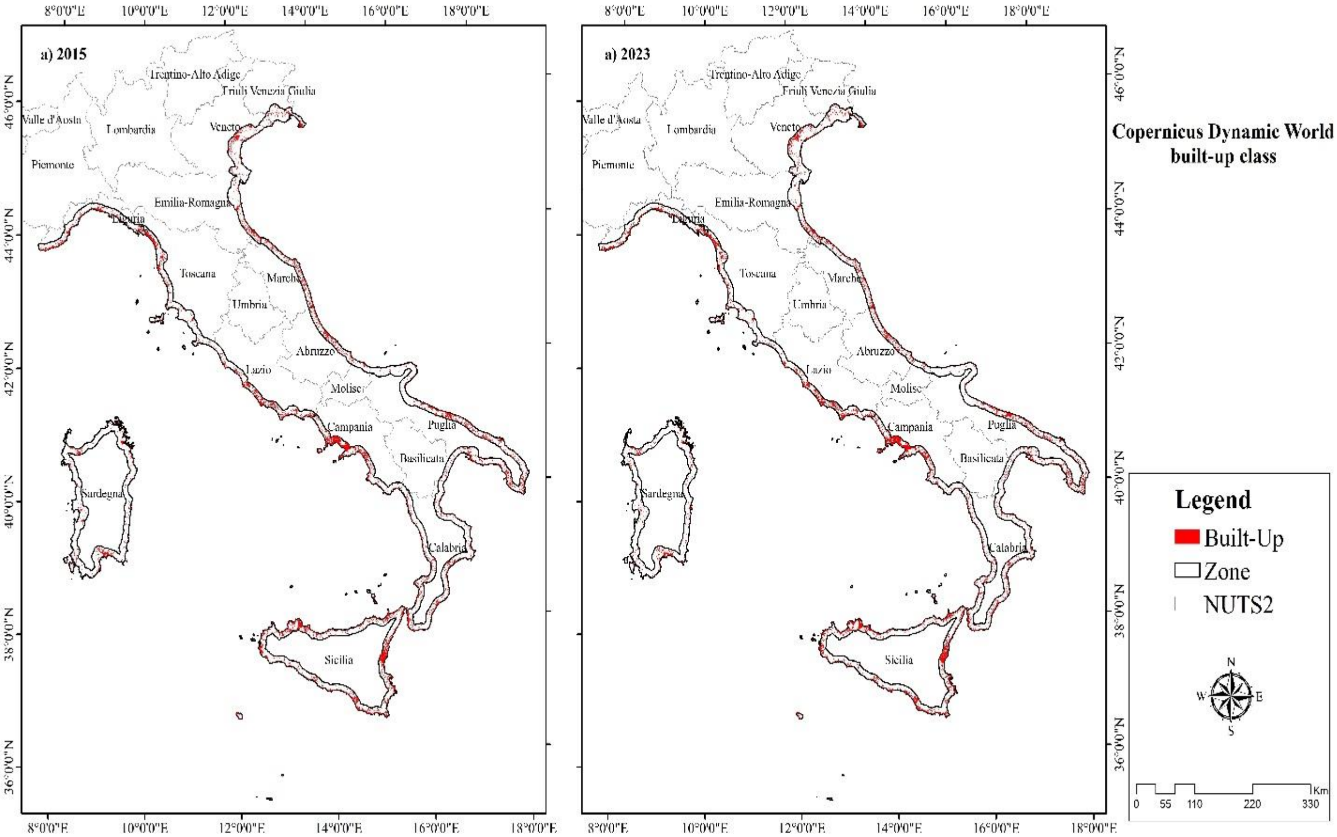

Fig. S1. Built-up class from Dynamic World - 10m global land cover dataset in Google Earth Engine created in ArcGIS Pro 3.3 (https://pro.arcgis.com/en/pro-app/latest/get-started/release-notes.htm) for years 2015 and 2023.

2. MK Test and Sen's Slope Estimator on monthly NTL

After obtaining access to the monthly data, we applied the MK Significance Trend Test for analysis. The results revealed a consistent upward trend in the total coastal zone, similar to the pattern observed in the summer season, with the exception of the central part. Furthermore, we noted a higher magnitude of the increasing trend (Sen's Slope value) in the southern part over the past decade (Fig. S2).

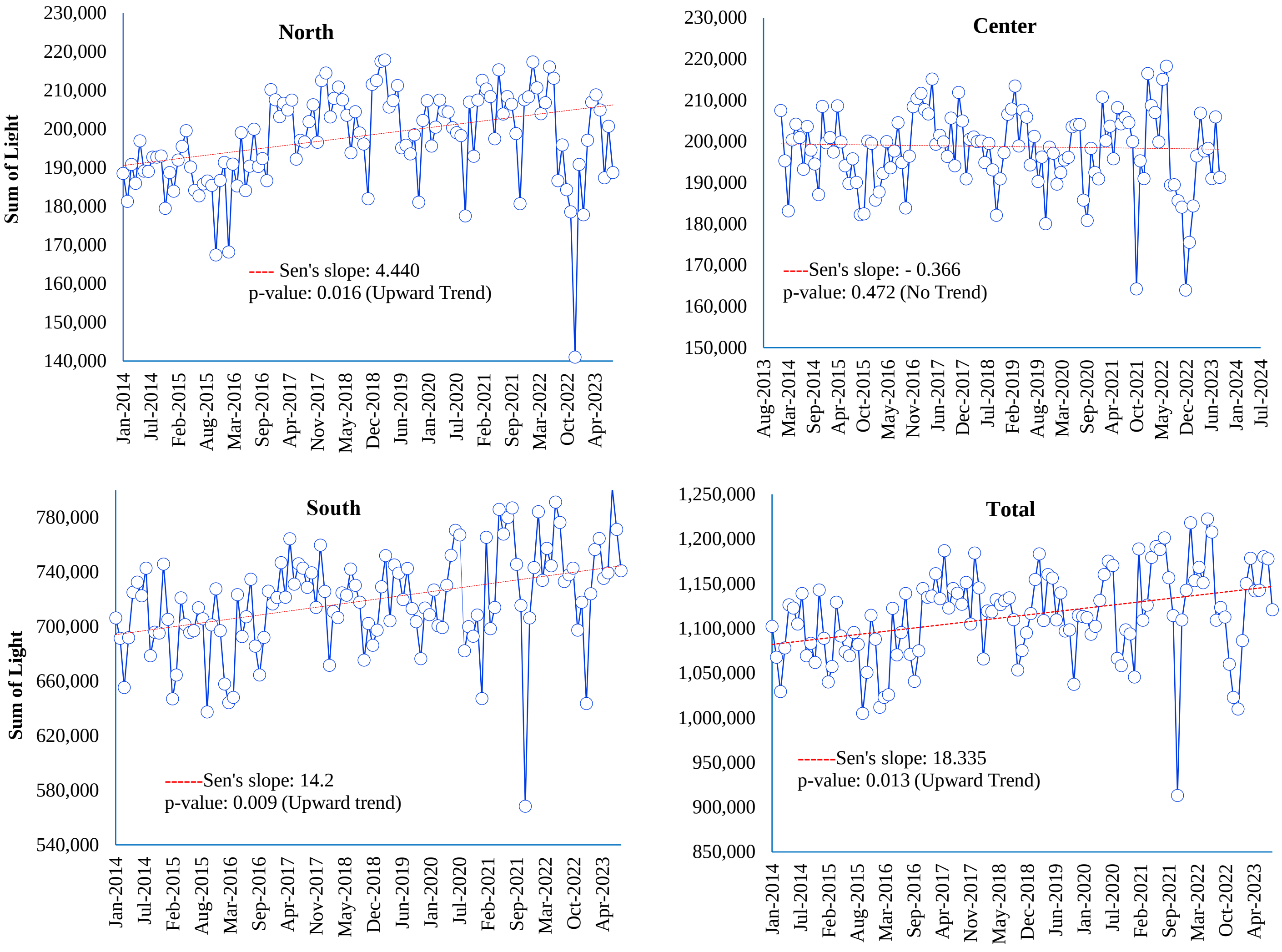

Fig. S2. Temporal change using Sen's Slope Estimator and Mann-Kendall Trend Test on monthly-based NTL (2014-2023) in northern, central, southern, and total coastal zones. *Dotted lines indicate the magnitude of trend based on Sen's Slope Estimator.
