# Supplementary material for: Nighttime light extent and intensity explain the dynamics of human activity in coastal zones
Source: Sci Rep. 2025 Jan 11;15:1663. doi: 10.1038/s41598-025-85917-z (PMC11724959; doi:10.1038/s41598-025-85917-z)
Supplement: Supplementary file 2 — Supplementary Material 2 [file 41598_2025_85917_MOESM2_ESM.docx]

**Nighttime light extent and intensity explain the dynamics of human activity in coastal zones**

Zahra Mokhtari^a^, Angela Stefania Bergantino^b^, Mario Intini^b^, Mario Elia^a^, Alessandro Buongiorno^b^, Vincenzo Giannico^a^, Giovanni Sanesi^a^, Raffaele Lafortezza^a*^

**Supplementary material**

1. **Built-up land cover change**

We obtained the Built-up cover for 2015 and 2023 from Dynamic World datasets. The extent of the built-up class expanded by 5% over this time frame.

**
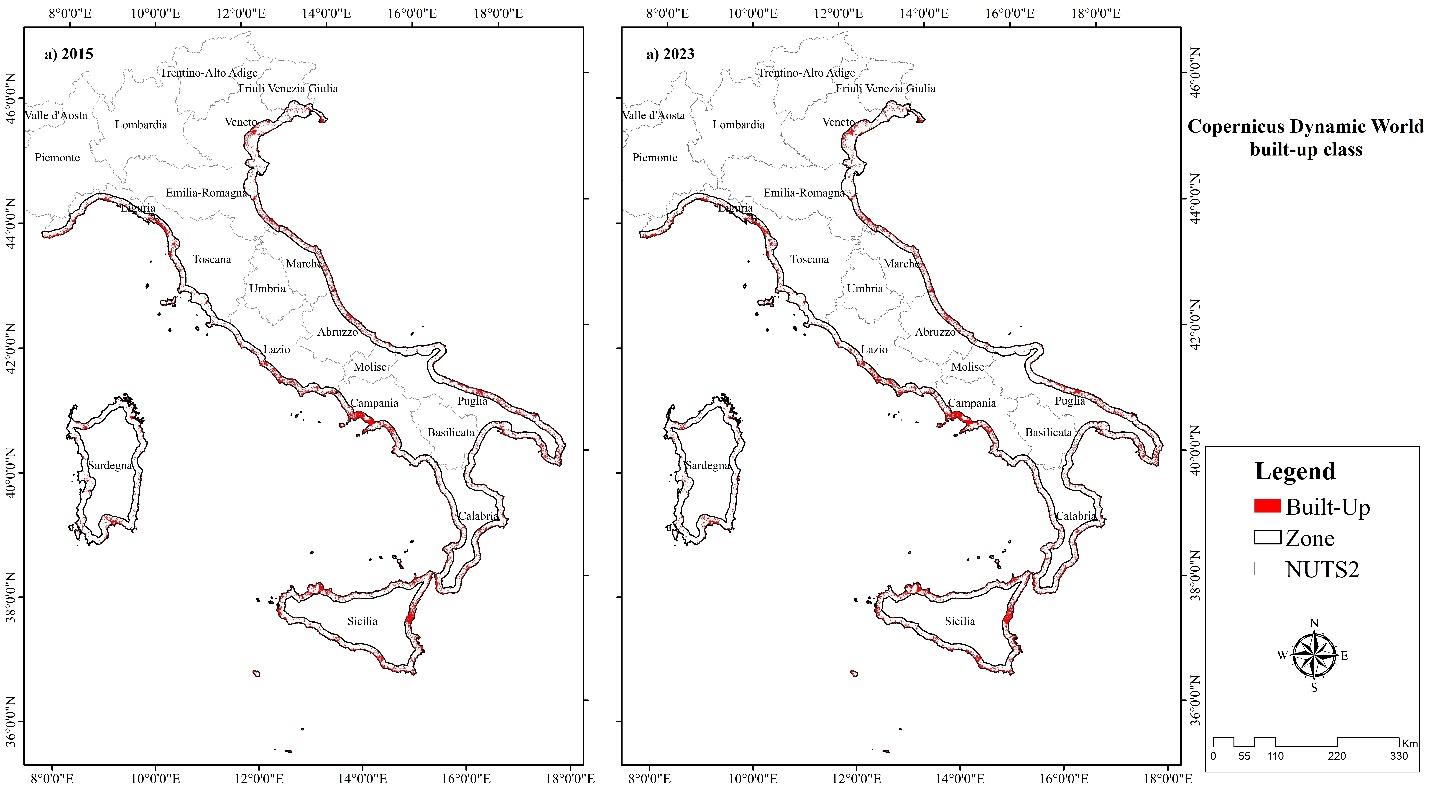
**

Fig. S1. Built-up class from Dynamic World - 10m global land cover dataset in Google Earth Engine created in ArcGIS Pro 3.3 (<https://pro.arcgis.com/en/pro-app/latest/get-started/release-notes.htm>) for years 2015 and 2023.

1. **MK Test and Sen’s Slope Estimator on monthly NTL**

After obtaining access to the monthly data, we applied the MK Significance Trend Test for analysis. The results revealed a consistent upward trend in the total coastal zone, similar to the pattern observed in the summer season, with the exception of the central part. Furthermore, we noted a higher magnitude of the increasing trend (Sen’s Slope value) in the southern part over the past decade (Fig. S2).

| **Sum of Light** |  |
| --- | --- |
| **South** |  |

Fig. S2. Temporal change using Sen’s Slope Estimator and Mann-Kendall Trend Test on monthly-based NTL (2014-2023) in northern, central, southern, and total coastal zones. *Dotted lines indicate the magnitude of trend based on Sen’s Slope Estimator.
